# Land use change and climate dynamics in the Rift Valley Lake Basin, Ethiopia

**DOI:** 10.1007/s10661-022-10393-1

**Published:** 2022-09-15

**Authors:** Ayenew D. Ayalew, Paul D. Wagner, Dejene Sahlu, Nicola Fohrer

**Affiliations:** Department of Hydrology and Water Resources Management, Christian-Albrechts-University, Kiel, Germany

**Keywords:** Land use dynamics, Random forest, Principal component analysis, Change point analysis, Climate dynamics, Rift Valley

## Abstract

Land use and climate dynamics have a pronounced impact on water resources, biodiversity, land degradation, and productivity at all scales. Thus, in this study, we present the spatio-temporal dynamics of land use change and climate aiming to provide a scientific evidence about gains and losses in major land use categories and associated drivers and significancy and homogeneity of climate change. To this end, Landsat images and historical climate data have been used to determine the dynamics. In addition, population census data and land use policy have been considered to assess the potential drivers of land use change. The spatio-temporal land use dynamics have been evaluated using transition matrix and dynamics index. Likewise, shifts in the climate data were analyzed using change point analysis and three homogenous climate zones have been identified using principal component analysis. The results show that, from 1989 to 2019, the areal percentage of agricultural land increased by 27.5%, settlement by 0.8%, and barren land 0.4% while the natural vegetation, wetland, water body, and grass land decreased by 24.5%, 1.6%, 0.5%, and 2.1%, respectively. The land use dynamics have been stronger in the first decade of the study period. An abrupt shift of climate has occurred in the 1980s. In the last four decades, rainfall shows a not significant decreasing trend. However, a significant increasing trend has been observed for temperature. Rapid population growth, agricultural expansion policy, and climate variability have been identified as the underlying drivers of land use dynamics.

## Introduction

The earth’s terrestrial surface and climate have been changed considerably over the past decades because of urbanization, deforestation, and agricultural expansion (Luyssaert et al., 2014; Winkler et al., 2021). In the past, recognition of the importance of the natural environment for human well-being has been less influential in maintaining sustainable development and poverty alleviation strategies (Jane et al., 2014; Vira, 2015).

In Rift Valley Lake basin, the economy is depending on small-scale rainfed cultivation. However, this low yield traditional cereal mono-cropping system did not fulfil the food demand of a rapidly growing population after the 1980s. As a result, farmers have recently intensified agriculture as a response to the pressure from food-deficit resulting from low yields (Garedew et al., 2009; Malmberg & Tegenu, 2007).

Several studies have been conducted to analyze the impact of climate change on water resources in the Rift Valley Lake basin (Alemayehu et al., 2006; Legesse & Ayenew, 2006; Awulachew et al., 2007; Van Halsema et al., 2011; Jansen et al., 2007). Gisbert (2010) investigated that most researchers understand that water resources have been diminished due to reduction of rainfall over the Rift Valley. Moreover, the decreasing in rainfall has been investigated in the study area (Kassie et al., 2014; Matewos & Tefera, 2020). However, a few scholars disagree on the reduction of rainfall over the last 50 years (Ayenew, 2004a; Gebrechorkos et al., 2019). These disagreements within the scientific community result in difficulties for decision makers and planners. Therefore, scientific evidences are required to understand the nature of climate and land use change dynamics in the Rift Valley Lake basin.

The study of land use and climate dynamics is critical for proper planning, management, and utilization of natural resources. Traditional methods, pixel-based (unsupervised and supervised classification), for gathering and analysis of geographic, demographic, and environmental data are not adequate for multi-complex environmental studies (Maktav et al., 2005), since many environmental issues exist that require handling multi-disciplinary data sets. Multi-spectral remote sensing data can be used to monitor the dynamics of land use (Lei et al., 2021; Saatchi et al., 1997; Thenkabail et al., 2005; Wagner et al., 2013) but is constrained by weather conditions, topographic complexity, and heterogeneity of LULC (Wagner et al., 2013; Wilken et al., 2017). Moreover, parametric classifiers like maximum likelihood (ML) depend on a predefined data model, and the performance of these classifiers depends on how well the data match the predefined model (Pal & Mather, 2004). Therefore, other non-parametric classification methods have been proposed to overcome this problem (Gislason et al., 2006; Benediktsson & Swain, 1992; Benediktsson, 1990). Among non-parametric classifiers, random forest (RF), support vector machines (SVM), and artificial neural networks (ANN) are the most common machine learning algorithms that are used to classify remote sensing data. Past studies have shown that remote sensing integrated with machine learning algorithms is a vital and efficient tool to develop and understand the biophysical change of the earth and to detect heterogenic land use (Steinhausen et al., 2018; Wang et al., 2006). Machine-learning algorithm classifiers often show a higher classification accuracy for mapping heterogeneous land use (Adam et al., 2014; Christovam et al., 2019; Gong et al., 2013). In particular, ensemble classifiers have been proposed (Benediktsson & Swain, 1992; Hansen & Salamon, 1990; Kuncheva, 2003). Random forests are a decision tree-type ensemble classifier. The algorithm is highly data adaptive (Bauer & Kohavi, 1999), and computationally less intensive. Therefore, random forest has been selected to detect land use change in this study.

Apart from land use change analysis, the spatio-temporal variability of climate has been evaluated aiming at detecting both climate and land use changes in the basin, as well as to derive scientific evidences for decision makers, water resources and land use planners, and other stakeholders. The basin is a monsoon-dominated region, where almost all agricultural communities rely on the monsoon summer rainfall for their livelihood. However, the increasing variability of rainfall and considerable change in the duration, intensity, and frequency of rainfall, rainfall periods (wet and dry spells), and onset and offset of rainfall have become a big concern. Change in the monsoonal rainfall characteristics, especially the variability of onset/ offset and wet/dry spells, and its adverse effect on agricultural activities have gained attention by agronomists and agricultural development agents, because the onset/offset (length of rain) and the wet/dry spells affect farmer’s crop and livestock. However, there is no scientific investigations of the rainfall characteristics in the basin. Moreover, most people, including the scientific community, perceived that the Rift Valley Lake basin has a homogenous climate regime. Also, climate and climate change impact studies in the basin have no common results, conclusions, and recommendations. Therefore, decision makers, water resources planners, development agents, agronomists, and other stakeholders are not able to take decisions, e.g., with regard to climate change adaptation, mitigation, and disaster risk reduction. Likewise, many recent studies indicate that there is a substantial and dynamic LULC change in the upper part of the basin (Desta & Fetene, 2020; Elias et al., 2019; Kidane et al., 2012), but there are no comprehensive scientific evidences of LULC change in the lower and central part of the basin. To address these research gaps, homogeneous climate regions, spatio-temporal variability for each homogeneous region, and the land use dynamics are analyzed.

## Material and methods

### Study area description

**Fig. 1:**
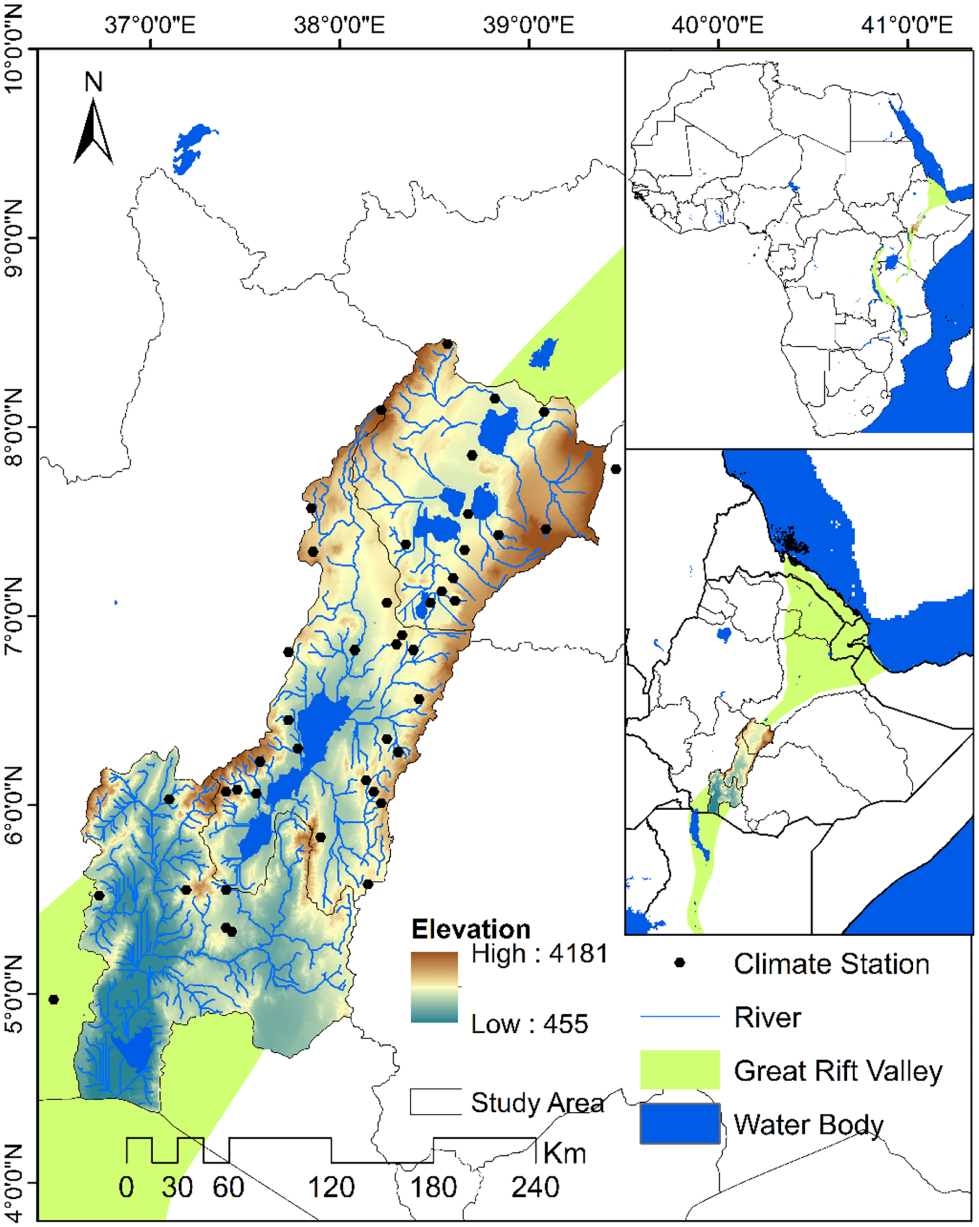
Ethiopian Rift Valley Lake basin

The Great Rift Valley (GRV) is a 4000-km-long fault line that stretches from the Red Sea to Mozambique’s and Zambia Valley. It is the single largest geographical feature in Africa and it transects the countries of Ethiopia, Kenya, Uganda, Rwanda, Burundi, Zambia, Tanzania, Malawi, and Mozambique (Gregory, 2018). The study has been conducted in the Ethiopian part of the Rift Valley Lake basin located between 36° and 40°E and 4° and 9°N. It extends from the Afar depression southwards to Kenya across the broad basins of Abijata-Ziway, Abaya-Chamo, and Segen (Fig. 1) and has an area extent of 55,050 km^2^.

The basin is characterized by a complex and rugged topography. Based on the digital elevation model (USGS) data, the valley floor altitude ranges from 500 to 1700 m above sea level and it is bounded by western and eastern escarpments with altitudes of up to 4181 m above sea level.

The basin contains lakes, wetlands, forests, grasslands, and savannah which provide a range of ecosystem services, e.g., as a natural habitat for a diversity of birds, fishes, and wildlife species. It contains over 500 bird species of African, European, Arabian, and Asian origin and 33 fish species (Sissay, 2003). In particular, many lakes of the region are a focal point for internationally important numbers of wildfowl and waders as well as rare or endangered species. In addition, Bird Life International has identified it as one of the important bird habitats in the world (Pascual-Ferrer et al., 2008).

### Data acquisition and processing

Time series of daily and monthly rainfall and temperature data for 44 stations have been used to evaluate spatio-temporal climate patterns and climate variability in the basin from 1951 to 2019. Landsat satellite data are widely used for LULC classification and analysis (Abdullah et al., 2015; Jiménez et al., 2018) due to their free availability and the good temporal and spatial coverage. In this study, post-monsoonal (23 January to 3 March), georeferenced Landsat images with a cloud coverage less than 10% have been employed, since satellite images are more likely to be affect by seasonal cloud cover from April to November. Since the study area is large, eight Landsat scenes are needed to cover it. Histogram contrast matching has been performed to mosaic these Landsat scenes together using histMatch function of the R package “RSToolbox” (Leutner et al., 2017). In addition, other ancillary data like the stream network, the basin boundary, the Ethiopia Basin boundary, the African country boundary, the Great Rift Valley boundary, a DEM, and water bodies have been obtained from the Ministry of Water Irrigation and Electricity (MoWIE). Ground truth information of land use has been collected during a field survey for the year 2019 and from Google Earth images for classification and accuracy assessment. Crop data has also been used to identify the type of crops and cropping system. Finally, population census data has been considered to evaluate the correlation between LULC change and rapidly growing population. The major data sets used in this study are presented in Table 1.

**Table 1:** List of acquired data used in this study

**Platform**	**Sensor**	**Resolution(m)**	**Acquisition date**	**Sources**
**Landsat-5**	TM	30	Feb 18, 1989	http://earthexplorer.usgs.gov
**Landsat-5**	TM	30	Feb 25, 1999	http://earthexplorer.usgs.gov
**Landsat-5**	TM+	30	Mar 03, 2009	http://earthexplorer.usgs.gov
**LANDSAT_8**	OLI	30	Jan 23, 2019	http://earthexplorer.usgs.gov
**Crop data**		-	2005/6–2019/20	CSA
**Climate**		-	1951–2019	NMA
**Population**		-	1987–2019	CSA

*OLI* Operational Land Imager, *TM* Thematic Mapper, *TM*+ Enhanced Thematic Mapper Plus, *NMA* Ethiopian National Meteorological Agency, *CSA* Ethiopian Central Statistical Agency

### Methodology

#### Flowchart of the methods

**Fig. 2:**
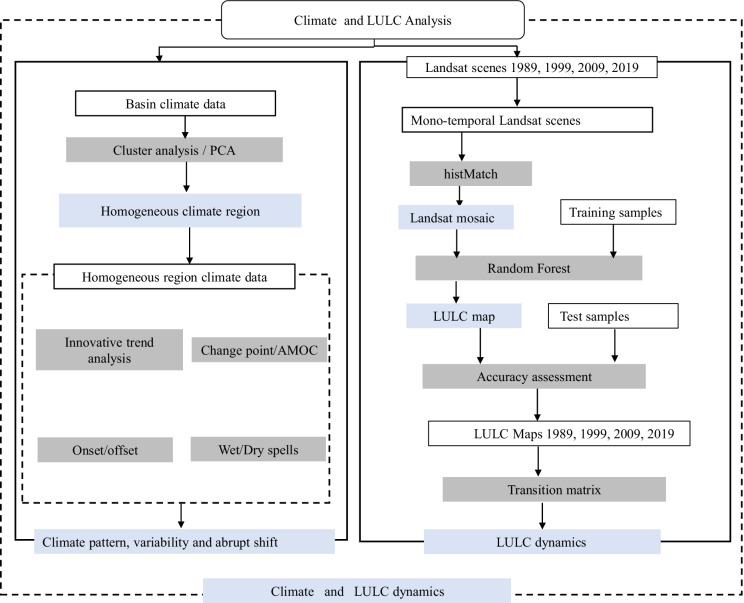
Flowchart of the methods

The general study design is shown in Fig. 2. All calculations have been carried out in the R programming environment, which is available from the Comprehensive R Archive Network (http://cran.r-project.org/). Random forest ensemble machine learning algorithms has employed for Land Use Land Cover (LULC) classification. Following the identification of homogenous climate region via cluster analysis, spatio-temporal climate patterns and variability have been evaluated using innovative trend analysis (ITA), and an analysis of change points, onset/offset of rainfall, and wet/dry spells.

#### Land use classification and accuracy assessment

The criteria for determining land use classes are not uniform throughout the world, and the definitions of classification types also differ among different classification systems (Zhang et al., 2011). In this study, the Food and Agriculture Organization Land Cover Classification System (FAOLCCS) is adopted to establish an effective link between remote sensing land use information and the literature (Table 2).

**Table 2:** Land use classes based on the FAO classification system

**Land use class**	**FAO classification system**
**Water body**	Water body: lakes, streams, canals, reservoirs, ponds
**Agriculture land**	Cropland, paddy field, cropland/other vegetation mosaic
**Wood land**	Scattered large trees
**Grass land**	Herbaceous, herbaceous with sparse tree/shrub
**Forest**	Broadleaf evergreen forest, broadleaf deciduous forest, needle leaf evergreen forest, needle leaf deciduous forest, mixed forest, tree open, mangrove
**Shrub**	Shrub, sparse vegetation
**Settlement**	Residential, commercial and services, industrial, transportation, communications, and utilities and built-up land
**Wetland**	Forested wetland and no forested wetland
**Barren land**	Bare area, consolidated (gravel, rock); bare area, unconsolidated (sand)
**Alpine**	Grasses, sedges, forbs, cushion plants, mosses, and lichens of alpine climate

Various classification methods have been developed to extract essential information from Landsat imageries. Since the launch of the first land observation satellite Landsat-1 in 1972, many classifiers, e.g., maximum likelihood, and, in recent years, machine learning algorithms have been used for classification. Among, pixel-based ensemble machine learning algorithm random forest (RF) has been selected for this study. Because of its versatility, RF has been used in a wide range of earth science applications including modeling of forest cover (Betts et al., 2017), spatio-temporal land-use modeling (Araki et al., 2018), land-cover classification (Nitze et al., 2015), wetland identification (Berhane et al., 2018; Maxwell et al., 2016), and object-oriented land use mapping (Kavzoglu, 2017). For this work, random forest (Breiman, 2001)-supervised classification approach was applied to produce land use classifications for the years 1989, 1999, 2009, and 2019 in R (Team et al., 2020). This algorithm uses decision trees and selects the best solution through voting/averaging. The number of trees required to maintain a certain level of accuracy has been assessed by several authors, and the minimum number of trees for optimal classification varies between 100 and 300 trees (Lawrence et al., 2006). For this study, depending on the quality of satellite images, several polygons have been created for each year. The number of polygons for the year 1989, 1999, 2009, and 2019 are 448, 294, 736, and 640, respectively. Polygons of each year have been split equally for training and testing sample and then, ten random points has been created for each training and testing polygons. Each classification has been carried out with 200 decision trees using the extracted spectral information from the training points. The accuracy of each year classification has been determined and evaluated with the test data using confusion matrices, overall, user’s, and producer’s accuracies (Story & Congalton, 1986).

#### LULC change dynamics

##### Land use transition matrix

To quantify the spatio-temporal land use dynamics, the classifications have been compared with the help of a transition matrix (Singh et al., 2017). The transition matrix consists of rows of previous land use categories (T1) and a column of recent land categories (T2) (Table 3). Aij denotes the land area that experiences transition from category i to category j. The entire diagonal indicates unchanged areas. The area of the land use in category i in time T1 (Ai+) is the sum of Aij over all j. Likewise, the area of the land use in category j in time T2 (A + i) is the sum of Aij over all i. In addition, the loss area has been determined as the difference between the row total and the persistence land use area (Ai+–Aii). Similarly, the gains have been calculated as the difference between the columns total and the persistence land use area (A + i–Aii).

**Table 3:** Land use transition matrix

**T2**							
		L1	L1	..	Ln	Ai+	Loss
**T1**	L1	A11	A11	…	A1n	A1+	A1+ – A11
	L2	A11	A22	…	A2n	A2+	A2+ – A22
	Ln			…	Ann	An+	An+ – Ann
	A + j			…	A + n		
	Gain			…	A + n–Ann		

##### Trend and rate of change

This study adopted the single dynamic index to evaluate the change of speed and trend of regional land use (Wang et al., 2001; Yi et al., 2013), and is defined as follows

1 $$\mathrm{K}=\frac{\left({\mathrm{A}}_{\mathrm{r}}-{\mathrm{A}}_{\mathrm{p}}\right)}{{\mathrm{A}}_{\mathrm{p}}}\times \frac{1}{\mathrm{T}}\times 100$$

where, ${A}_{r}\text{ and }{A}_{p}$ are the area of a certain land use category at the end (recent) and beginning (past) of the research period, respectively, and T is the number of years between the two land use maps.

Also, the transfer rate among land use categories for the research period has been evaluated using the integrated land use dynamic index (Qingqing et al., 2012) that reflects the overall change of all land categories formulas as follows:

2 $${K}_{total}=\frac{{\sum }_{1}^{n}\left|{A}_{ri}-{A}_{pi}\right|}{2\sum_{1}^{n}{A}_{pi}}\times \frac{1}{T}\ x\ 100$$

where, ${A}_{ri}\text{ and }{A}_{pi}$ are the area of a certain land use category of the recent and past land use map, respectively, *T* is the year difference between the recent and past land use maps, and *n* is the number of land use categories.${ K}_{total}$ is the annual integrated change index of area change of all land use categories.

#### Climate patterns and change point analysis

##### Climate dynamics and homogeneous regions

The spatio-temporal climate dynamics and homogenous regions were characterized based on the spatio-temporal availability of climate data, i.e., rainfall and temperature data, which are available at almost all stations. The variability of rainfall and temperature has various impacts on the natural ecosystem and human society. Consequently, many climatological studies are based on the analysis of rainfall characteristics (Rao & Hada, 1990; Rao et al., 2016; De Oliveira et al., 2014) and temperature (Da Silva, 2004; Vincent et al., 2005). The spatial pattern and variability of rainfall and temperature over the Rift Valley Lake basin have been investigated for the 44 years of data (1950–2019) using principal component analysis (PCA). In particular, it is an objective partitioning method and widely used across various research studies for cluster analysis to identify homogeneous rainfall regions (Oliveira-Júnior et al., 2017; Teodoro et al., 2016; Terassi & Galvani, 2017). The concepts and the algorithms used to execute a cluster analysis with PCA are inherently different. The PCA can be computed either using eigendecomposition or singular value decomposition (SVD). Since our data matrix is huge, it was hard to compute the eigenvalues of the covariance matrix using eigendecomposition, which sometimes causes round-off errors. However, singular value decomposition (SDV) is a stable computational method often used to compute PCAs of a dataset by truncating the less important basis vectors in the original SVD matrix. It involves a reduction of data dimensionality and is primarily used to explain mutual dependence and variance of huge series of data (Praus, 2005). Among other statistical tools, SVD is considered a simple, precise, and robust method for dealing with the correlation between fields and the preferred method for numerical accuracy (Bretherton et al., 1992). It extracts the most significant modes of variability in comparison to other statistical techniques (Wallace et al., 1992). The dimension of the data is reduced by projecting it into the space spanned by the principal components (PCs). To that end, we calculate the eigenvectors of the variance matrix via SVD. The data were centered and scaled to explain the amount of variation (Becker et al., 1988) using the open-source statistical software R.

##### Climate change point analysis

An abrupt climate change occurs when the climate system is forced to cross some threshold, thereby triggering a transition to a new state at a rate that is determined by the climate system itself and which is faster than the cause (Alley et al., 2002). In the field of data mining, abrupt change can be seen as a sudden change in a data series. Statistical analysis has proven to be a useful technique to detect climatic patterns and changes in the past (Khapalova et al., 2013). Change-point analysis is a widely used statistical approach to detect the change points in a data series that are defined as a point at which the parameters’ mean, variance, or trend of a distribution change abruptly (Beaulieu et al., 2012; Vu et al., 2019). It has been shown to have the potential to detect abrupt shifts (Zhao et al., 2012). Recently, especially in earth system sciences, change-point analysis has been applied to detect changes in temperature (Croitoru et al., 2012, Shirvani, 2017), precipitation (Khapalova et al., 2013; Xie et al., 2018), and changes of the river water level (Wong et al., 2006). Therefore, for this study, change point analysis has been chosen as analytic tool to detect abrupt shift and dynamics in 44 years of rainfall for region II (central part of study area) and region III (southern part of study area) and 66 years of rainfall for region I (northern part of study area) and 55 years of temperature datasets. Popular algorithms for change point analysis as implemented in the R package strucchange (Zeileis et al., 2002), breakpoints (Zeileis et al., 2002), and Bayesian (Wang et al., 2018) were tested in this study. Break and Bayesian change point analysis have been found very suitable for our dataset. Break point analysis has been used to detect the optimal positioning and number of change points for the dataset. The critical change point in mean has been accessed using at most one change point (“AMOC”) method (Hinkley, 1970) with the cpt.mean() function and the dynamics of the time series have been detected using Bayesian change point analysis method (Sung et al., 2017).

##### Onset and offset of rainfall

To identify the onset and offset of the wet season, we apply a method proposed by Liebmann et al. (2012), which is widely applicable for rainfall seasonality analysis across Africa. As described in Liebmann et al. (2012), initially, the start (d_i) and end (d_f) of the wet season have been computed from the annual cumulative of the dataset. Secondly, the onset and offset have been determined from daily cumulative rainfall anomaly in the range of the window (d_i − 30 to d_f + 30), where the minimum (maximum) daily cumulative rainfall was considered as onset and offset. The value 30 refers to the maximum shift of onset and offset in days. A moving window was used to extract the start and end of rainfall from the time series data using onset package in R (Becker et al., 1988).

##### Wet and dry spells

Wet and dry spells have been extracted from the baseline period (1983–2018) to evaluate the space and time variability of wet and dry spells. The simplest way to define the onset of the wet spell would be to find the first wet day with rainfall above a given threshold (Lau & Yang, 1997; Liebmann & Marengo, 2001, Nieto‐Ferreira & Rickenbach, 2011) and considering the persistence of precipitation and the occurrence of dry spells or wet spells. This method agrees well with the planting in rain-fed agriculture (Marteau et al., 2011; Tadross et al., 2005). In this study, a wet spell is defined as three consecutive days with precipitation greater than 1 mm, and a dry spell is defined as three consecutive days with precipitation less than 1 mm (Bichet & Diedhiou, 2018; Froidurot & Diedhiou, 2017).

## Result and discussion

### Land use classification and accuracy assessment

The accuracy assessment for the four land use maps is shown in Table 4. The overall accuracies of 92.0, 93.3, 93.1, and 94.9% for the years 1989, 1999, 2009, and 2019, respectively, indicate excellent classification accuracy for the majority of land use classes, indicating that the land use maps are suitable for the subsequent analysis and change detection as proposed by Pontius (2000). User accuracies range from 83.5 to 99.7%.

**Table 4:** Quality of the land use classifications

		LANDSAT 5 TM		LANDSAT 5 TM +	LANDSAT8 OlI
Indicators	Classes	1989	1999	2009	2019
Overall accuracy		92.3	93.3	93.1	94.9
User accuracy	Barren land	91.2	95.8	94.7	95.0
	Agriculture	89.6	94.7	97.67	96.2
	Alpine	87.7	97.5	93.8	97.1
	Forest	96.3	98.0	96.3	96.7
	Grass	93.3	83.5	93.3	91.1
	Settlement	84.9	86.6	90.0	89.2
	Shrub	96.7	92.5	91.9	93.3
	Water	99.7	98.8	99.7	97.8
	Wetland	85.8	94.3	93.3	94.7
	Woodland	98.2	89.1	83.7	94.3

The classification has been validated using ground truth obtained from a field survey (2019) and google earth image (1986–2019). This showed that the water class was better distinguished and grass class was poorer distinguished than other classes. The water bodies, mainly lakes, are large water areas which are usually easy to detect. Whereas, grass was difficult to distinguish from agricultural land. The high level of classification accuracy for these classes could be attributed to the large number of training samples, selected remotely sensed data, dry season, and image-processing (contrast adjustment and histogram equalization) and classification approach.

The resulting land use maps of the four years 1989, 1999, 2009, and 2019 shown in Fig. 2 have overall ten land use classes. Most parts of the basin area are used for agriculture land. Agricultural production is dominated by mixed farming.

Cereal-cereal crop rotation is the usual practice in the rift valley, but sowing pulses preceding to cereals (like maize-haricot bean rotation) and inter-crops (cereal with pulse) are becoming more common (Burayu et al., 2006). A 2015/2016 crop and livestock production report of CSA (2016) showed that the irrigated agriculture is mainly vegetable and fruit production predominantly practiced along the rivers and lakes. Different types of crops are being grown in the basin including grain crops, cereals, ensete (false banana, Ethiopian banana, pseudo-banana), root crops, fruits, coffee, chat, sugarcane, oilseeds, vegetables, and pulses. Ensete is the dominant crop followed by grain crops, cereals, and root crops.

Semi-natural vegetation is the second most dominant land use class of the basin characterized by dry evergreen mountain forest, evergreen shrub, and Acacia woodland. It varies distinctively with the altitude, and above 3000 m, the dominant vegetation type is afro-alpine and sub-afro-alpine ericaceous shrub and dispersed clumps of highland bamboo (*Arundinaria alpina*) around valley bottoms and springs. The basin also contains eight major lakes which lie in the Rift floor and are fed by large perennial rivers originating from either side of the mountainous ridges. The largest of these lakes is Lake Abaya (1137.82 km^2^) that accounts for 36.8% of the water body area. Other major lakes include Lake Chew Bahir (462.1 km^2^, 14.9%), Lake Zway (415.9 km^2^, 13.4%), Lake Chamo (307.7 km^2^, 9.94%), Lake Shale (300.4 km^2^, 9.7%), Lake Langano (229.16 km^2^, 7.4%), Lake Awassa (91.7 km^2^, 3%), and Lake Abijata (68.4 km^2^, 2.2%).

The individual class percentage area change statistics of each class in the four periods are summarized in Table 5 and show that agricultural area had the largest share in all periods of the total land use categories followed by vegetation (forest, woodland, shrub, and alpine). Except agriculture, settlement, and barren land, other land use classes faced a major decline during the last three decades. The study also revealed that there was about 27.5% areal percentage increase of agricultural area, 0.4% barren, 0.8% settlement at the cost of 24.5% vegetation, 1.6% wetland, 0.5% water body, and 2.1% grassland from 1989 to 2019. These values signify the rapid land use change driven by agricultural expansion that exerts pressure on non-agricultural areas, in particular vegetation lands and wetlands. Expansion of the already existing agricultural land through rapid deforestation of woodland, shrub land, grass land, wetland, and forest units all combined together led to its continuous expansion throughout the basin. Shrubland and forest land are the two sub-classes that have been most impacted, losing 14.9% and 7.8%, respectively, from the total land area in the last three decades.

**Table 5:** Temporal quantitative change of land use

Class	Area (%)				Change (%)
	1989	1999	2009	2019	1989–2019
Agriculture	30.89	45.60	54.00	58.40	27.51
Alpine	1.09	1.00	1.00	1.10	0.01
Barren	1.48	1.00	1.60	1.90	0.42
Forest	11.19	7.50	3.70	3.40	−7.79
Grass	4.60	7.90	4.70	2.50	−2.1
Settlement	0.02	0.20	0.40	0.80	0.78
Shrub	30.87	19.10	18.80	15.90	−14.97
Water	6.08	5.80	5.00	5.60	−0.48
Wetland	2.20	0.60	0.30	0.60	−1.6
Woodland	11.58	11.30	10.50	9.80	−1.78

### LULC change dynamics

**Fig. 3:**
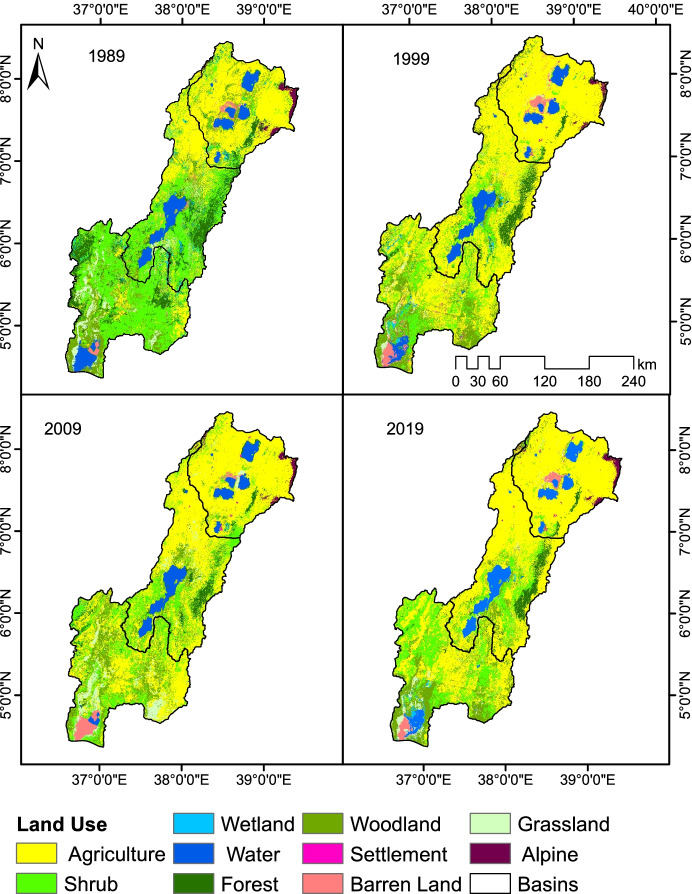
Spatio-temporal distribution map of land use

The spatial land use dynamics presented in Fig. 3 show maximum changes in the first and second decade of the study period. Spatially, the dynamics were particularly strong in the southern part of the basin. Traditionally, grazing was dominant in this part of the basin, so that there was no more cultivation practice before 1990. However, after 2000 cultivation, practices have become common in the area due to rapid population growth and the community adopt crop cultivation systems. As a result, the land use change is very strong in this region in all periods. In addition, land use change is observed in the central part Abijata-Ziway and Nechisar National Parks due to agricultural expansion, illegal settlement, and tree cutting for fuel.

The land-use dynamic index during the periods of 1989–1999, 1999–2009, and 2009–2019 is shown in Table 6. It underlines that there was a strong land use change in the first decade of the study period. The maximum land use dynamic index was in settlement, up to 148.5% followed by wetland (−7.4%), grassland (7.0%), agriculture (4.8%), and shrub land (−3.8%). For most classes, land use dynamics were the strongest in the first decade. Only settlement and water had the maximum index in the last decade (2009–2019). The integrated dynamic index (K) value was 1.8, 0.5, and 0.1 for time period (1989–1999), (1999–2009), and (2009–2019), respectively, and confirms that the land use change was strongest for the first decade and decreased in the latter two decades.

**Table 6:** Speed and trend of land use change

**Class**	**Single dynamic index (%)**		
	**K (1989–1999)**	**K (1999–2009)**	**K (2009–2019)**
**Agriculture**	4.8	0.9	0.8
**Alpine**	−0.8	−0.2	0.7
**Barren land**	−3.3	3.0	−0.4
**Forest**	−3.3	−2.6	−0.2
**Grass land**	7.0	−2.0	−2.9
**Settlement**	75.3	95.0	148.5
**Shrub**	−3.8	−0.2	−0.7
**Water**	−0.6	−0.6	1.0
**Wetland**	−7.4	−2.7	1.6
**Woodland**	−0.2	−0.1	−0.2

Furthermore, the overall temporal dynamics of land conversion (“from-to”) has been evaluated. The land transfer index (Table 7) shows that the majority of the land use has been changed into agriculture between 1989 and 2019. The maximum land transformation was observed in wetland as only 0.6% of its original cover remained unchanged. Water, alpine, agriculture, and settlement have relatively high shares of persistent land use (> 65%).

**Table 7:** Transfer matrix of land use

	**2019**										
**1989**	Class	Agriculture	Alpine	Barren	Forest	Grass	Settlement	Shrub	Water	Wetland	Wood
	Agriculture	90.6	0.7	0.3	0.4	0.9	0.3	3.0	0.1	0.2	3.7
	Alpine	26.0	66.2	0.0	0.6	0.1	0.00	6.7	0.0	0.0	0.2
	Barren	30.9	0.0	20.8	0.2	7.5	1.2	9.6	14.7	0.4	14.7
	Forest	44.0	0.4	0.1	19.4	0.6	0.1	28.1	0.9	0.6	5.9
	Grass	45.9	0.0	1.1	0.3	18.7	0.4	8.8	0.1	0.9	23.8
	Settlement	0.0	0.0	0.0	0.0	0.0	99.3	0.8	0.0	0.0	0.0
	Shrub	49.2	0.2	0.2	3.1	1.2	0.1	31.6	0.3	0.3	13.9
	Water	0.8	0.0	11.4	0.0	3.7	0.0	0.2	82.5	0.3	1.1
	Wetland	73.1	0.3	0.1	4.6	0.3	0.2	15.6	1.2	0.6	4.1
	Wood	52.9	0.0	0.6	0.2	3.4	0.7	9.4	1.0	2.8	29.2

### Climate pattern and abrupt shift detection

#### Construction of homogeneous climatic regions

**Fig. 4:**
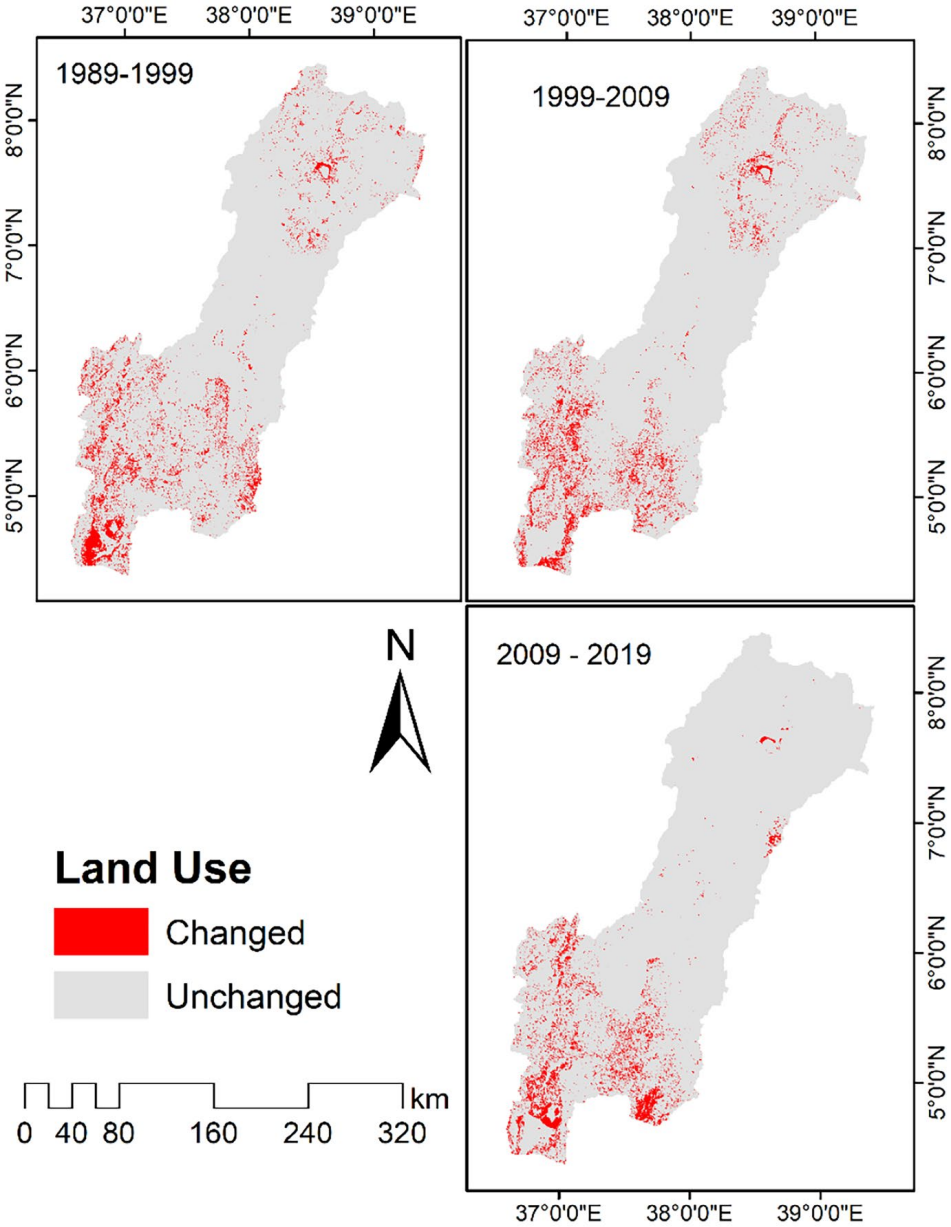
The spatial dynamics of land use change show stronger changes in the southern part of the study area

The principal component analysis (PCA) suggests three main homogenous rainfall regions and three main rainfall regimes (Fig. 4). The vector lines grouped together show a high correlation between weather stations and are considered as a homogeneous climate zone. The points show the temporal variability at a monthly time scale. Region I is the upper part of the basin which is formed by 11 stations, region II is the central part of the basin which is formed by 15 stations, and region III is the lower part of the basin and formed by 18 stations according to their pertinence degrees.

**Fig. 5:**
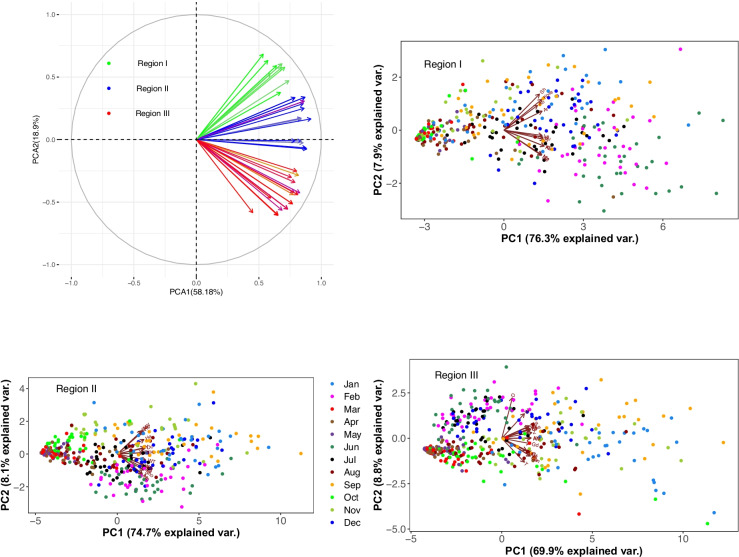
Spatio-temporal variability of rainfall. The PCA plot of the first two PCs of the data set explains 80% of the variation, colored by month of the respective data

**Fig. 6:**
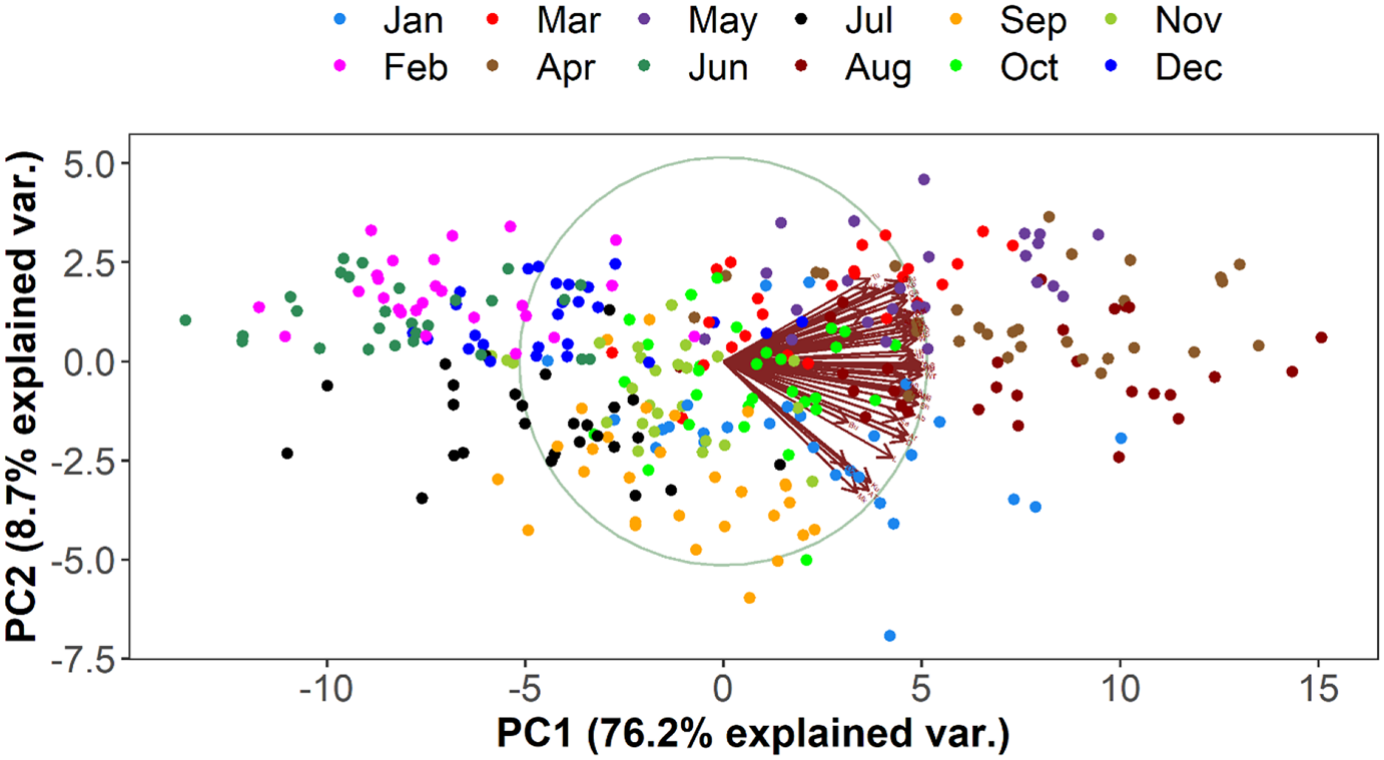
Spatio-temporal variability of temperature: The PCA plot of the first two PCs of the data set explains 80% of the variation, colored by month of the respective data

The first two PCs are explaining more than 80% of the total variability in the dataset (Figs. 5 and 6). They have a standard deviation of the principal components greater than 1 providing a good incite of the variability of rainfall and temperature. The rainfall variability is high during the transition periods, i.e., spring and autumn. Likewise, the PCA shows that there is a high temporal variability of temperature. Especially, its variation is very high during the wet season and is mainly associated with changes of cloud cover.

**Fig. 7:**
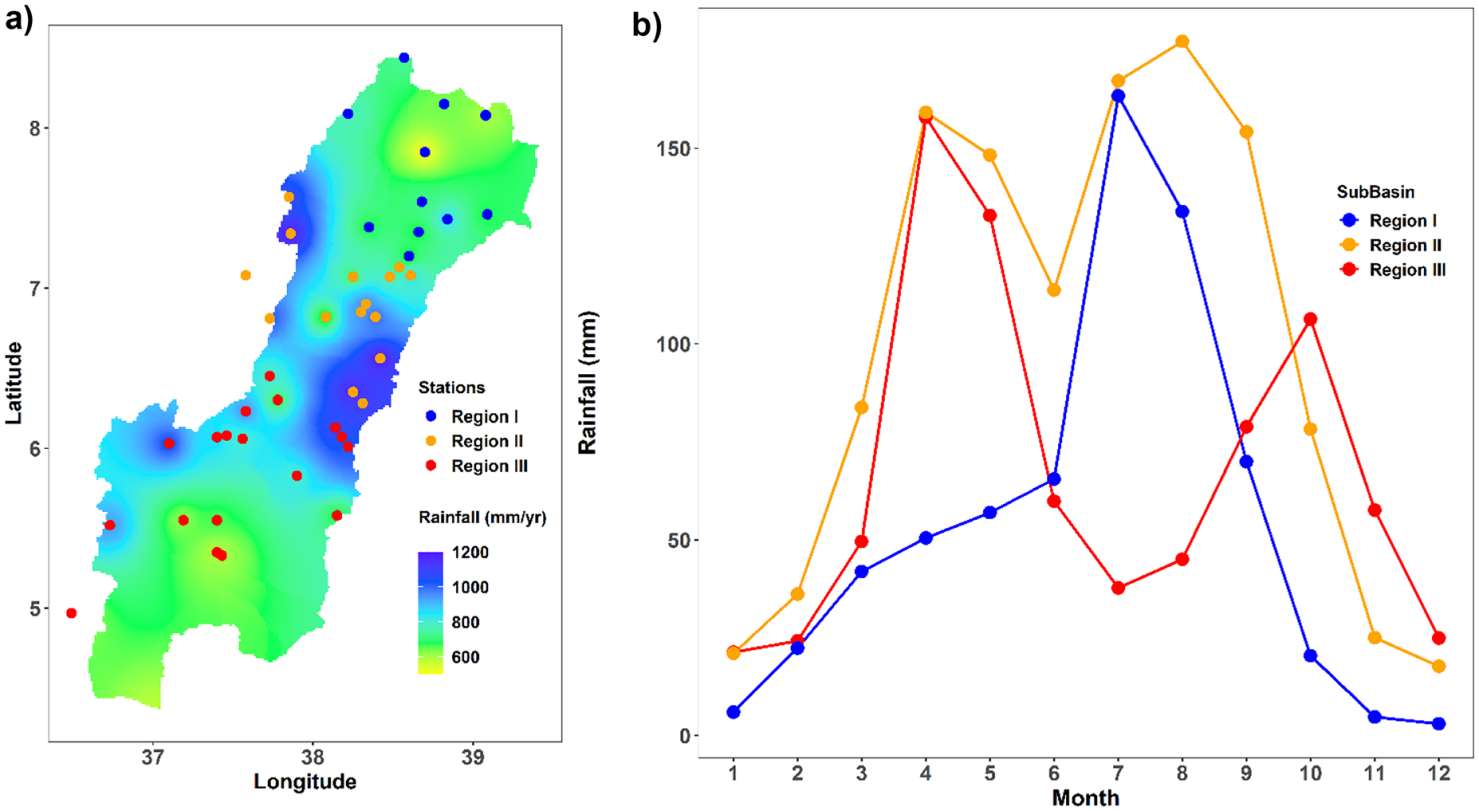
Rainfall of the homogeneous regions of the Rift Valley Lake basin: **a** the spatial distribution of rainfall over the basin interpolated using inverse distance weighting (IDW); **b** the temporal pattern of rainfall for each region

**Fig. 8:**
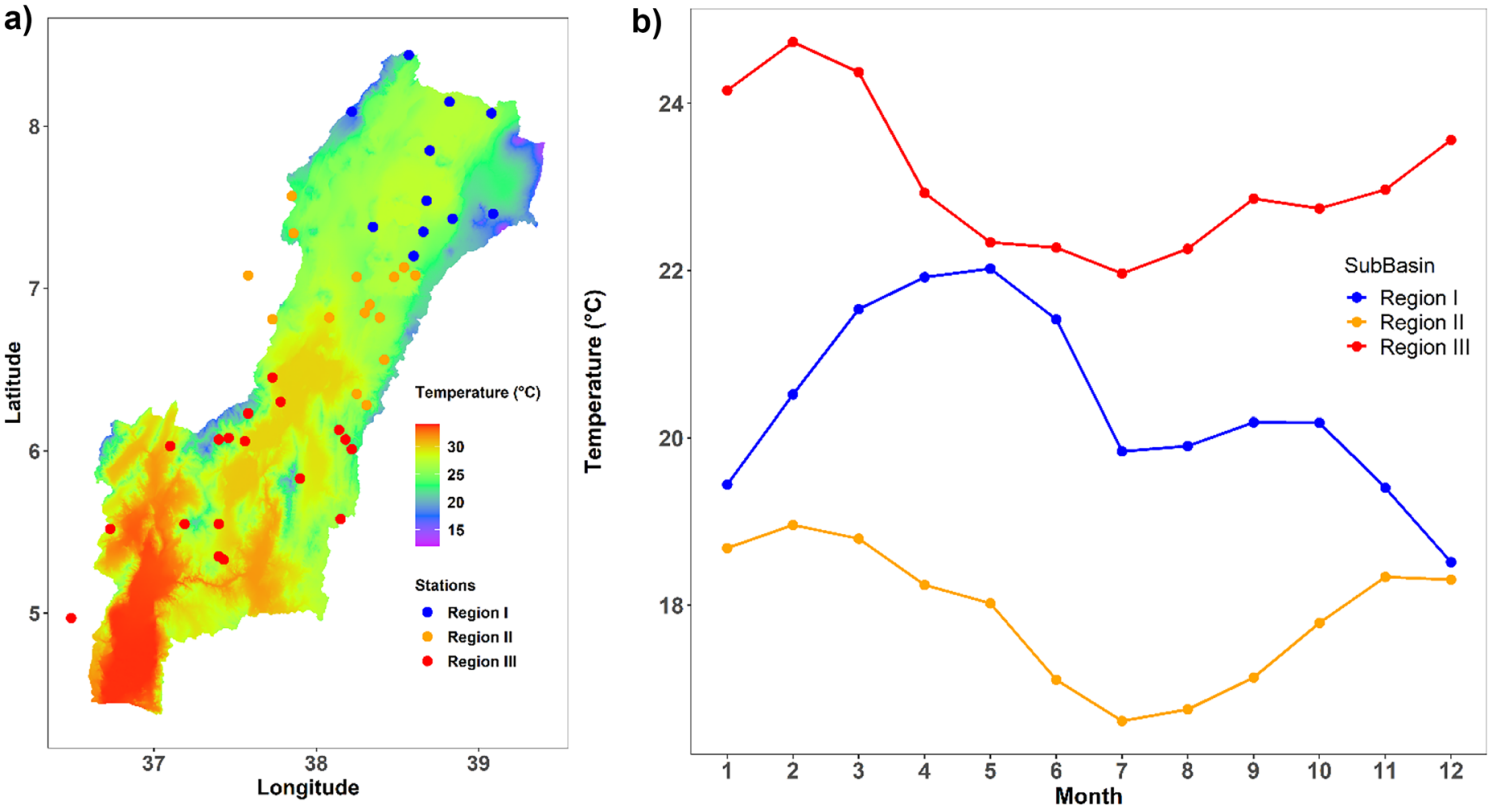
Temperature for the homogeneous regions of the Rift Valley Lake basin: **a** the spatial distribution of long term annual average temperature; **b** the annual average temperature cycle obtained from the available weather stations

As can be seen in Figs. 7 and 8, the rainfall and temperature patterns over the basin are heterogeneous in space and time. Region I is characterized by lower volumes of annual average precipitation ranging from 400 to 860 mm with one peak in August. Region II is characterized by a higher volume of annual precipitation ranging from 704 to 1200 mm with a bimodal climate pattern with peaks in April and August. Likewise, region III is characterized by a bimodal rainfall pattern with peaks in April and October. It receives an average annual precipitation ranging from 500 to 1100 mm with maximum rainfall in April. Three seasons can be distinguished based on precipitation for regions I and III and two seasons for region II. Region I has summer (June–September), spring (March–May), and winter (October–February) seasons. Similarly, region III has spring (March–July), summer (August–November), and winter (December–February). However, region II has a long summer (March–October) with a little break in June and a winter season (November–February).

Distinct patterns in the seasonal temperature were also identified (Fig. 8). The intra-annual, inter-seasonal as well as the diurnal temperature variability is very high in the entire basin. The temperature also varies widely throughout the basin, from the hottest on rift floor to frost-prone in the afro-alpine zone. The maximum seasonal average temperature is occurring during dry season for all climate zones. This variation from 10 to 36 °C is mainly due to differences in elevation and cloud cover. The southern part of the rift is lower, warmer, and drier than other parts of the basin.

#### Climate pattern and variability

##### Trend analysis

**Fig. 9:**
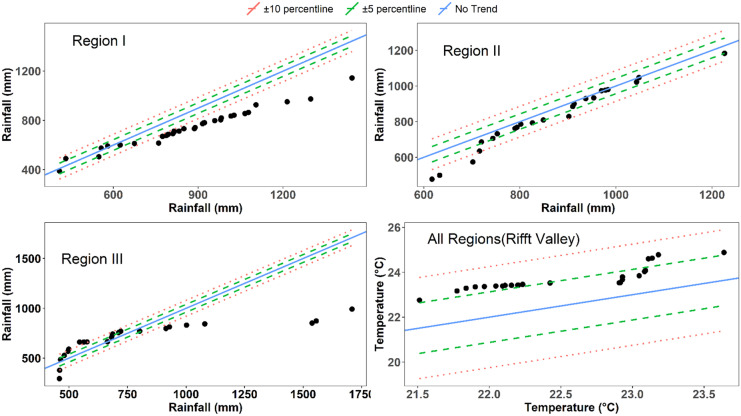
Innovative trend analysis of climate over the Rift Valley Lakes basin

The trend of an annual total rainfall and temperature series has been evaluated for the study area (Fig. 9). The ITA method has been applied to the annual rainfall time series of the three regions and to the annual average temperature time series of the whole study area. ITA provided visual inspection with quantitative trend slopes distinctively for low, medium, and high sub-values of the time series with physical interpretations. With regard, rainfall records have insignificant decreasing trends in low, medium, and high values for region I and region II and insignificant increasing trends in low value of region III. In contrary to rainfall, temperature records have significantly strong increasing tendency in low, medium, and high values along the basin.

As a confirmation of the results of the ITA method, the significance of the trend has been verified with the Mann–Kendall test. The *p*-value of the rainfall data was larger than 0.05 (ranging from 0.125 to 0.514). Therefore, with regard to the 5% significance level, no significant trend in the rainfall data can be identified. However, the *p*-values for temperature show a highly significant trend with *p*-values below the standard 0.05 (ranging between 2.38e^−07^ and 0.00096).

##### Climate dynamics and change point detection

**Fig. 10:**
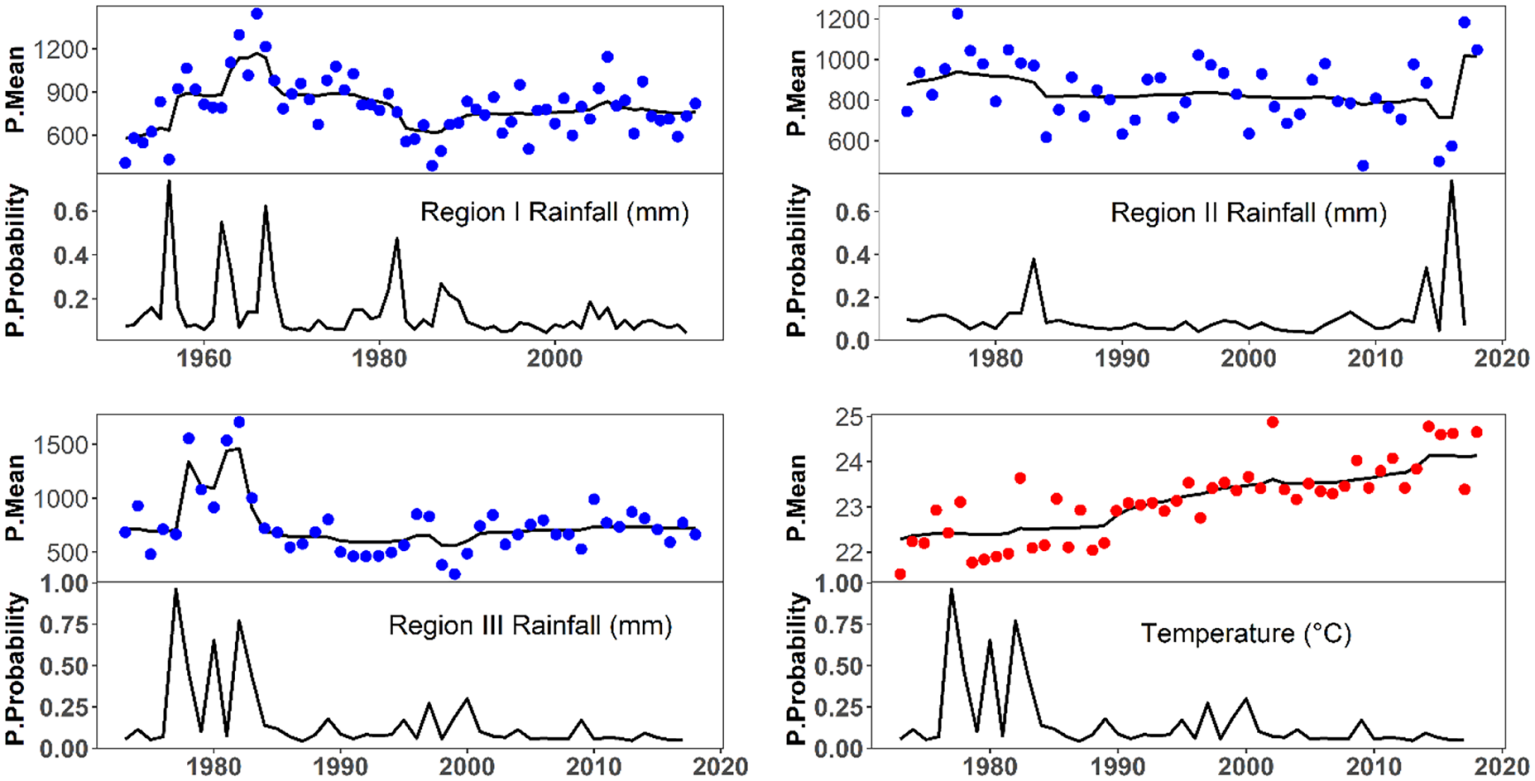
Change point detection (1970–2018): estimated posterior mean (P. Mean) displays the data along with the posterior means of each position and posterior probabilities (P. Probability) display the proportion of iterations resulting in a change point at each position

The change point analysis presented in Fig. 10 shows that there is an abrupt shift in rainfall and temperature over the Rift Valley Lake basin. The change point analysis results revealed that rainfall has shifted downward after the year 1983. Apparently, the rainfall of region I was very dynamic from the year 1954 to 1970 with maximum probability of 0.784 of a change in mean and posterior means. And the climate was stable from 1970 to 1980 and again the region has experienced dynamic change from 1981 to 1992 and becomes stable after 1992. Likewise, region II has experienced a decrease after a change point from the 1980s until 2015 and more recently, a large increase was observed. A moderate dynamic variation was observed before the change point as well. In region III, after a change point in the mid-1980s, decreased posterior mean values have been maintained. Before 1983, the variation was very dynamic. In summary, the rainfall variation was highly dynamic before the 1980s and became more stable after the middle of the 1980s.

Likewise, a change point analysis has been performed for temperature for the entire basin, and the result revealed that an abrupt upward shift has occurred in the 1980s. The posterior mean value of temperature was stable before the 1980 and from 1990 to 2010. However, from 1980 to 1990 and after 2010, it was very dynamic. The dynamics of temperature in the basin was mainly associated with the length of wet season and cloud cover. In fact, sunny days prevail during most of the year in almost the entire basin. The alternation of cloudy and clear sky conditions results in ample temperature differences and the annual temperature amplitude is limited and seems to be controlled by the intertropical circulation and the monsoon pattern rather than by elevation (Fazzini et al., 2015).

##### Onset and offset of the rainy season

**Fig. 11:**
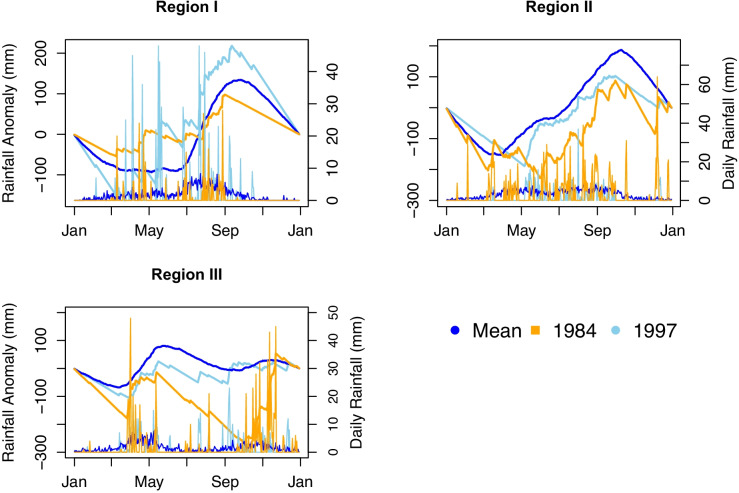
Onset and offset analysis across Rift Valley basin using the daily cumulative rainfall anomaly based on a 30 days moving window

The result for the analysis of onset and offset of the rainy season is presented in Fig. 11. Along with the mean of the 36-year-long record, we show the maximum and minimum difference to the mean to visualize the variability of onset and offset from the mean. Thus, the long-term mean onset of the rainy season in region I is in the second week of June and the rainy season ends in the last week of September. The onset is varying from mid-May to the end of June and the offset is varying from the first week of September to the first week of November.

Likewise, the onset of rainfall in region II varies from the second week of March to the last week of April and the offset is varying from the last week of September to the last week of October. Its long-term mean onset is in the third last week of March and the offset is in the last week of October. It has a long rainy season with a short break in June. In region III of the basin, the first onset is varying from first week of February to the first week of April and the offset is varying from the second week of May to the last week of June. The second onset is varying from the last week of August to mid-October and the offset is varying from the second week of September to the last week of November. The second long-term mean of onset and offset is the last week of September and the third week of November, respectively. However, the long-term mean of onset and offset of the first rainy season is mid-March and end of May, respectively. The long-term mean of onset and offset of the second wet season of region III is the last week of September and the end of November, respectively. Both the onset and offset of the basin show insignificant decreasing trends. These reveal that the rainfall starts earlier and lasts earlier, i.e., the wet season is shifting.

##### Wet and dry spells

**Fig. 12:**
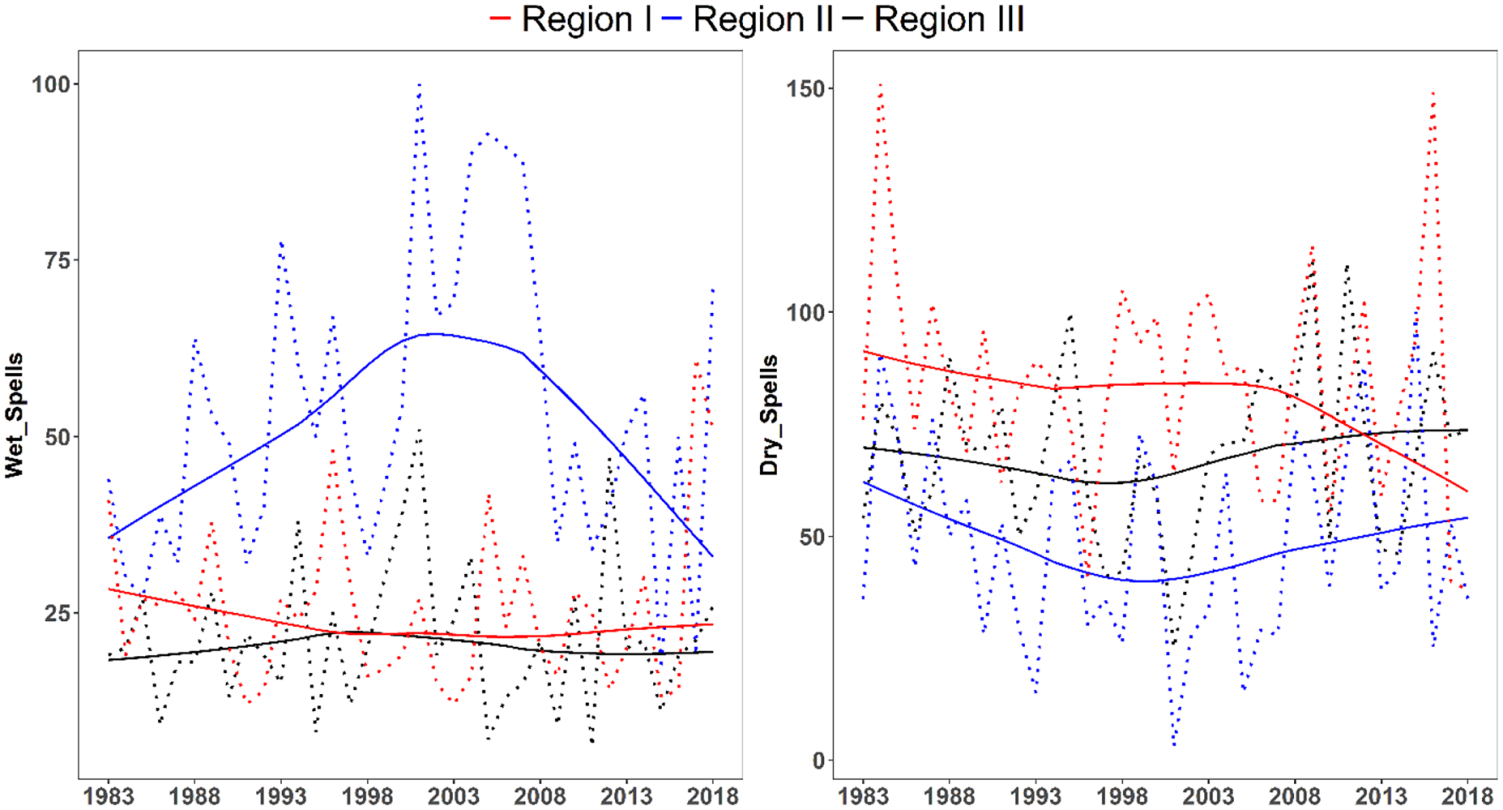
Wet and dry spells in all regions of Rift Valley basin

The temporal patterns of the wet and dry spells in wet season of each region of the Rift Valley basin are shown in Fig. 12. The highest number of dry and wet spell days were observed in 1984 at region III (151 days) and in 2001 at region II (100 days), respectively. Region II experienced the longest wet spells compared to the other regions and region I experienced the longest dry spells. There is high spatio-temporal variability of dry and wet spells across the basin (Fig. 12). The Mann–Kendall trend test indicates that no significant changes have been found for both wet and dry spells in all regions of the basin, as the *p*-values of wet and dry spells are larger than 0.05 (ranging from 0.219 to 0.836).

The variation of wet and dry spells is mainly associated with south-northward migration of the ITCZ resulting in longer wet spells in region II and long dry spells in region I.

## Discussion

The rapid expansion of agriculture and decline of vegetation for the first two decades is mainly linked to the civil war between the socialist Dergue regime and Ethiopian People Republic Democratic Front (EPRDF) in 1990/1991. During the war and after the fall of the Dergue regime, people used the natural vegetation land illegally for agriculture and settlement. In addition, after the present government took power in 1991, it has embarked upon a new national land policy and agricultural expansion land use policy that promotes farming and the development of rural infrastructure aimed to raise food production and ensuring national food security (ARD, 2004). This has led to rapid reduction of the natural vegetation cover and rapid increase of agricultural land during the last three decades. Moreover, harvesting of trees for firewood and charcoal is another important activity reducing tree density of vegetation cover. Pronounced urban area increase resulted mainly from migration of people from rural to urban areas (Bundervoet, 2018) due to continuing ecological destruction, drought, famine, and war (Ezra, 2003) and the lack of necessary social infrastructures. An alarming decline of surface water areas in region I of the basin can be linked to high abstraction of water from the lakes and the tributaries feeding the reservoirs for irrigation and industry (Jansen et al., 2007; Legesse & Ayenew, 2006). Especially, the National Economic Development Strategy (NEDS) emphasizes the need for the agricultural sector to enhance food self-sufficiency, ensure food security at the household level, and for agriculturally led industrial development-aggravating agricultural expansion. Due the fact that the subsistence agricultural system cannot assure food security, the government supported the development of irrigation systems that have higher water use efficiencies. In this regard, the government has put forward more than 31,000 ha small- and large-scale potential irrigation development projects in the basin. All these together put pressure on water resources in the basin. Contrarily, the area coverage of water resources has increased in region III of the basin. This might be due to high surface runoff and sediment load associated with deforestation, and/or linkages of groundwater flow. Scientific evidences showed that the Ethiopian Refit Valley is an active tectonic and seismic region (Chorowicz, 2005; Ring, 2014; Saria et al., 2006, 2014; Stamps et al., 2008). This active tectonic movement faults might have created regional linkages of groundwater flow. However, this subject requires further investigation.

Apart from population growth and national land policy, high inter-annual climate variability has impacts on land use dynamics especially on water and wetland. The surface area of water and wetland has been decreased noticeably in the land use map of 2009, which is mainly associated with a reduction of annual rainfall in 2008/2009 and water abstraction, e.g., for irrigation and industrial water use (Ayenew & Tilahun, 2008; Jansen et al., 2007).

The onset, offset, wet spells, and dry spells are varying across the basin. Three homogenous climatological regions can be identified. The spatio-temporal variations of rainfall in the Rift Valley Lake basin are due to variations of topography and the north–south ward movement of the ITCZ. The ITCZ oscillates annually between a southward location of 15°S in January and a northward location of 15°N in July (Diro et al., 2011b). The ITCZ arrives between the first week of February and the first week of April in region III and will persist until end of May. Likewise, it arrives to region II from second week of March to last week of April and persist until the last week of September to the last week of October. The ITCZ continues to move north ward and arrives in region I between mid-May and the end of June and persists until the first week of September to the first week of November. After the first week of September to the first week of November, it starts to move back southward (Diro et al., 2009; Lashkari & Jafari, 2021a; Korecha & Barnston, 2007). This gives rise to the bimodal rainfall pattern in region II and region III and the monomodal pattern in region I of the basin. The variation of onset and offset window is depending on the weakening and strengthen of subtropical high-pressure systems (Dyer et al., 2022; Lashkari & Jafari, 2021b). Various previous studies have investigated that the Ethiopian summer rainfall is affected by the variation of equatorial pacific sea surface temperature (SST) and its teleconnection with summer rainfall (Block & Rajagopalan, 2007; Diro et al., 2011a; Gissila et al., 2004; Gleixner et al., 2017; Korecha & Barnston, 2007).

Apart from the spatio-temporal climate variability over the basin, also an abrupt climate shift has been identified in the 1980s. Many scientists have identified abrupt shifts of climate worldwide and the mechanisms behind the climate shift (Dansgaard et al., 1993; Johnsen et al., 1992; Broecker, 2006; Clement & Peterson, 2008; Marotzke, 2000; Overpeck & Cole, 2006) and the implications of abrupt change for the future (Alley et al., 2003; Palmer et al., 2011). They have investigated the possible reasons for abrupt changes include global warming takes, melting of ice caps and glaciers, increased precipitation and other inflows of fresh water to the North Atlantic Ocean that weaken or shut down thermohaline circulation, resulting in cooling of the North Atlantic Ocean. This change in ocean circulation could disrupt the transfer of moisture eastward to Ethiopia, where the study area is located. Another possible explanation of the change point is the changing vegetation cover over the study area. The role of natural vegetation and savanna grass change on modifying the climate at local as well as at global level has been well documented (Da Silveira Lobo Sternberg, 2001; Holm et al., 2014; Sud & Fennessy, 1982). Therefore, rapid decline of vegetation cover might have led to the abrupt shift of climate over the basin. Also, insignificant decreasing rainfall trends and significant increasing temperature trends have been observed in the basin. These findings are in agreement with other studies in the basin and in other basins in Ethiopia (Ayenew, 2004b; Kassie et al., 2014; Tigabu et al., 2020; Viste et al., 2013). Future climate change in the basin may alter the wet and dry soil conditions, length of winter and summer, and crop yield, i.e., it may affect LULC patterns in the basin. Moreover, as climate is a significant driver of land degradation processes, future climate change can result in further LULC change (Smith et al., 2020; Sunderland & Rowland, 2019).

## Summary and conclusion

The land use pattern has changed significantly between 1989 and 2019 in the Rift Valley Lake basin. The agricultural land maintained the largest area share, followed by vegetation and water body. Moreover, the agricultural, barren land, and settlement share of land increased by 27.5%, 0.4%, and 0.8%; vegetation land, wetland, grass land, and waterbody decreased by 24.5%, 1.6%, 2.1%, and 0.5 respectively from 1989 to 2019.

The analysis of the land use dynamic degree showed that the dynamics of wetland change was the largest during 1989–2019 followed by grass land and vegetation. Water areas, agricultural land, and settlement area were persistent land use classes. These changes can be related to sustained and ongoing population growth. The rapid population growth associated with a dependency on rural economy contribute to the ongoing land use changes. Deforestation, overgrazing, agricultural expansion, and water body declination become the emerging issues of the basin.

Water bodies are substantially declining in upper part of the basin because of high abstraction for irrigation, industrial, and other uses. However, in the lower part of the basin, the water bodies are increasing. The water area dynamics index (k) for the last decade (2009–2019) showed positive values. This is due to the appearance of a new lake near to south of Lake Chamo with a surface area of 10.45 km^2^. In addition, the expanding of area of Lake Abaya and Chamo has its own contribution. Even though further investigation is required, this study also thought the increasing of surface runoff and sediment load associated with deforestation has big contribution. Secondly, few studies have investigated that the major flow systems and groundwater flux into the lakes are controlled strongly by rift faults (Ayenew, 2001). Therefore, the increasing of water in the lower part of the basin might be linked with active tectonic movement faults and groundwater movement.

In addition to agricultural expansion, cutting tree for fuel and illegal settlement are also the main cause for deforestation of the basin. Apart from population growth and national land policy, highly inter-annual variability of rainfall has impacts on land use dynamics especially on water and wetland areas. Increasing agricultural production to meet rapidly growing food demand through strength agricultural intensification policy while safeguarding vital ecosystem services and promoting social equality lies at the heart of sustainable development.

The Rift Valley Lake basin is characterized by three climate zones and regimes and its summer(June–September) climate pattern controlled by several climatological features in the lower and upper troposphere (Hastenrath, 2012; Li & Hsu, 2018). These include the seasonal northward movement of the ITCZ, persisting over Ethiopia; formation of heat lows over the Sahara and Arabian landmasses; establishment of subtropical high pressure over the Azores, St. Helena, and Mascarene; southerly/southwesterly cross-equatorial moisture flow from the southern Indian Ocean, central tropical Africa, and the equatorial Atlantic; upper-level TEJ flowing over Ethiopia; and low-level jet (Somali jet) (Fekadu, 2015; Segele et al., 2009). The upper part (region I) of the basin has a mono-modal rainfall pattern with low rainfall amount relative to other parts of the basin, while the middle (region II) and lower part (region III) of the basin have bi-modal rainfall patterns. The rainfall is highly variable both in space and time but its change is not significant. However, change of temperature is significant and highly variable both in space and time. Furthermore, climate variability is explained via onset, offset, wet spell, and dry spell of rainfall. All of them are highly variable within the window, and the window is varying from region to region in the basin. Currently, there is no well-organized onset and offset information dissemination system for farmers and other users. Since many crop seeds will damage if they wait more days in the soil and farmers obliged to sow two or three times during late of onset. Not only, knowing of the length of wet season is really important to select type of crop to grow. Very common farmers grow the same crop type each year based on their own indigenous knowledge, rather considering the onset and offset information of rainfall. This might have high impact on agricultural system and yield.

The change point analysis also well investigated the abrupt shift of climate, occurred in the 1980s, across the basin. It also clearly identified the dynamic nature of climate over the basin. The abrupt shift and dynamicity of climate are linked with change in global ocean–atmosphere circulation (Hastenrath, 2012; Li & Hsu, 2018; Nicholson, 2019).

Developing adequate land use policy frameworks, enforcement regulations, and land use management plans could be effective to reduce the impact of land use change on ecosystem services. Diversification of climate adaptation strategies could be more effective in coping up climate variability impacts as the study area consists of various climate zone. To this end, the findings of this study, which shows the spatio-temporal dynamics of land use and climate change, could greatly enhance the awareness of concerned decision makers to devise relevant strategies for mitigation and adaptation of future climate and land change impacts in the basin.
